# Letter from the Editor-in-Chief

**DOI:** 10.19102/icrm.2017.080408

**Published:** 2017-04-15

**Authors:** Moussa Mansour


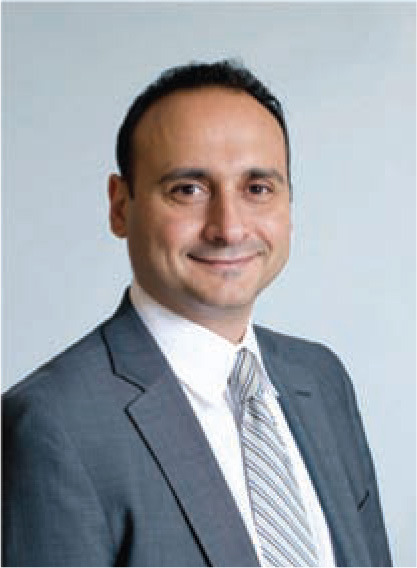


Dear Readers,

Patients with congenital heart disease are living longer into adulthood because of advances in corrective surgery. The congenital lesions in these patients often lead to hemodynamic overload at older ages, with subsequent cardiomyopathy and increased risk of sudden cardiac death. As a result, the number of patients with congenital heart disease receiving implantable cardiac pacemakers and defibrillators is rapidly growing. The management of these patients is challenging because they remain underrepresented in clinical studies, and might not fit into the established guidelines that apply to patients in the general population. Studies and publications focusing on this particular patient population are needed in order to advance our understanding of the pathology and improve our clinical management.

This issue of the *Journal* contains an important article by Grubb et al. titled “Pacemaker and Defibrillator Implantation in Patients with Transposition of the Great Arteries.” In this article, the authors describe the characteristics and clinical course of 63 patients with transposition of the great arteries with systemic right ventricles who received implantable cardiac devices. This article provides important information regarding the survival patterns of these patients, and a detailed description of defibrillator therapies.

Most of the treatment guidelines for patients with congenital heart disease are derived from the extrapolation of findings of studies involving other patient populations. Very few studies to date have targeted patients with complex congenital heart disease exclusively. As a result, the management of these patients continues to be challenging. Furthermore, the optimal strategies for risk stratification for sudden cardiac death remain controversial. Techniques and indications for catheter ablation for complex arrhythmias in this group of patients also remain poorly defined. Prospective clinical studies are needed in order to clarify all of these uncertainties, and help facilitate an improvement in the clinical care of this growing patient population.

I hope that you find the content of this issue educational and helpful to your practice.

Sincerely,


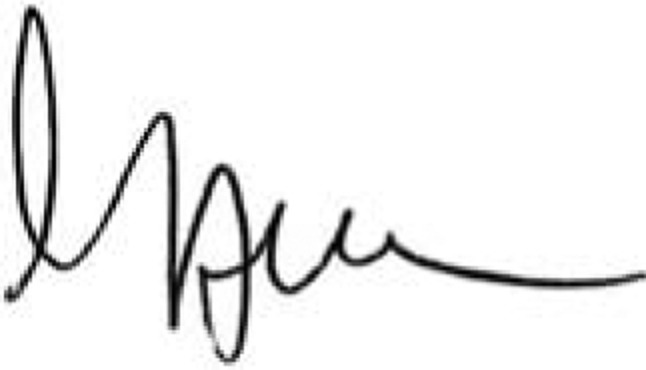


Moussa Mansour, MD, FHRS, FACC

Editor-in-Chief

The Journal of Innovations in Cardiac Rhythm Management

MMansour@InnovationsInCRM.com

Director, Cardiac Electrophysiology Laboratory

Director, Atrial Fibrillation Program

Massachusetts General Hospital

Boston, MA 02114

